# Supplementary material for: Content-rich biological network constructed by mining PubMed abstracts
Source: BMC Bioinformatics. 2004 Oct 8;5:147. doi: 10.1186/1471-2105-5-147 (PMC528731; doi:10.1186/1471-2105-5-147)
Supplement: Additional File 5 — The original Chilibot query results of the term "long-term potentiation (LTP)" and 22 other terms, limiting the latest references analyzed to the years 1990, 1995, 2000, and 2004. [file 1471-2105-5-147-S5.bz2 › chilibotAdditionalFile5/ltp2000/html/ATF_ACTIN.html]

 


 **ATF** and **ACTIN** 
  
Found 4 abstracts in PubMed,  **4 abstracts were retrieved and analyzed**.  


---

 Search Google  |
 PDF files only 
|  EDU domain only 

---

**Interactive relationship** (e.g. stimulation, inhibition, etc)

- A significant increase in the expression of the mRNA of tubulin and  **actin**  was observed in the brain of the  **aTf**  treated animals.  Ref: 10905629 Neurochem Res, 2000
- The aim of this work was to clarify if the accelerated myelination produced by  **aTf**  could be linked to changes in certain cytoskeletal elements present in the myelin fraction such as tubulin,  **actin** , and different microtubule associated proteins MAPs.  Ref: 10905629 Neurochem Res, 2000
